# Vitamin A Positively Correlates with Secretory Immunoglobulin A: A Cross-Sectional Study in Omicron COVID-19 Outpatients

**DOI:** 10.3390/jcm13061538

**Published:** 2024-03-07

**Authors:** Francisco Javier Turrubiates-Hernández, Samuel García-Arellano, Laura Elena Herrera-Jiménez, Guillermo González-Estevez, Fabiola Márquez-Sandoval, Diana Lourdes Padilla-Bórquez, Hazael Ramiro Ceja-Gálvez, Natali Vega-Magaña, Ferdinando Nicoletti, Guillermina Muñoz-Ríos, José Francisco Muñoz-Valle

**Affiliations:** 1 Instituto de Investigación en Ciencias Biomédicas, Centro Universitario de Ciencias de la Salud, Universidad de Guadalajara, Guadalajara 44340, Mexico; francisco.turrubiates@cucs.udg.mx (F.J.T.-H.); samuel.garcia4566@academicos.udg.mx (S.G.-A.); laura.herrera3967@alumnos.udg.mx (L.E.H.-J.); guillermo.estevez@cucs.udg.mx (G.G.-E.); diana.pborquez@alumnos.udg.mx (D.L.P.-B.); hazael.ceja2291@alumnos.udg.mx (H.R.C.-G.); alejandra.vega@academicos.udg.mx (N.V.-M.); 2 Doctorado en Ciencias de la Nutrición Traslacional, Centro Universitario de Ciencias de la Salud, Universidad de Guadalajara, Guadalajara 44340, Mexico; yolanda.marquez@academicos.udg.mx; 3 Laboratorio de Diagnóstico de Enfermedades Emergentes y Reemergentes, Centro Universitario de Ciencias de la Salud, Universidad de Guadalajara, Guadalajara 44340, Mexico; guillermina@academicos.udg.mx; 4 Department of Biomedical and Biotechnological Sciences, University of Catania, 95123 Catania, Italy; ferdinic@hotmail.com

**Keywords:** vitamin A, retinol-binding protein, secretory IgA, antibodies, COVID-19, SARS-CoV-2, respiratory tract infections

## Abstract

**Background:** Respiratory tract infections remain among the leading causes of mortality worldwide. The COVID-19 pandemic has highlighted the importance of mucosal immunity in defending against infectious agents. Vitamin A is known to influence the production of secretory immunoglobulin A (SIgA) predominantly in the gut, where it is a critical component of the first line of defense on mucosal surfaces. **Methods:** This cross-sectional study, conducted 14 days post-positive COVID-19 diagnosis, aimed to determine the relationship between the nutritional status of vitamin A and SIgA levels in COVID-19 outpatients. Serum and saliva samples were collected. Vitamin A nutritional status was determined based on the assessment of dietary intake and the analysis of retinol-binding protein 4 (RBP4). SIgA levels were analyzed from salivary samples. In addition, serum antibodies were analyzed. **Results:** Dietary vitamin A intake and RBP4 levels positively correlated with SIgA. Patients with higher vitamin A intake showed higher SIgA/IgG1 and SIgA/IgG3 ratios, while those with higher RBP4 levels showed higher SIgA/IgM, SIgA/IgG1, and SIgA/IgG2 ratios. **Conclusions:** These findings underscore a significant correlation between vitamin A nutritional status and SIgA levels in COVID-19 outpatients, which may suggest the potential importance of maintaining optimal vitamin A levels for the prevention of viral infections.

## 1. Introduction

Respiratory tract infections (RTIs) are the main cause of primary care visits. Although upper RTIs generally cause mild symptoms, they can occasionally lead to severe complications. However, lower RTIs are responsible for a significant number of deaths, ranking fourth among the leading causes of mortality worldwide [1,2,3]. More than three years after identifying the respiratory virus responsible for COVID-19, officials declared the end of the health emergency [4,5]. Nevertheless, SARS-CoV-2 remains worrisome because of its tendency to mutate into variants with augmented transmission capabilities, and it may pose a threat to evading immunity triggered by vaccines or past infections [5].

Due to the main transmission route of SARS-CoV-2, the immune response of the respiratory mucosa is crucial in the defense against infectious agents, where secretory immunoglobulin A (SIgA) is produced in considerable amounts [6,7]. In this sense, it has been suggested that targeting the production of SIgA may be an effective strategy for preventing respiratory infections [8,9,10].

Vitamin A is a fat-soluble compound acquired through the ingestion of foods abundant in retinol, retinol esters, or carotenoids [11]. Its metabolism results in the production of retinoic acid (RA), which when bound to its nuclear receptors acts as a transcription factor that regulates the expression of different genes, some of which serve functions within the immune system. Vitamin A plays a critical role in mucosal immunity, where RA has been documented to contribute to the class switch to IgA in gut-associated lymphoid tissues (GALTs) [11,12].

Since the recognition of the relevance of vitamin A in treating measles, researchers have investigated the close connection between vitamin A deficiency and respiratory infection incidence [13,14]. Preclinical studies have shown that Wistar rats which were fed a vitamin A-deficient diet had lower SIgA concentrations than those in the control group [15]. Similarly, mice that were intranasally inoculated with influenza virus and provided a vitamin A-rich diet or vitamin A supplements had higher concentrations of SIgA compared to the control group [16,17,18].

On the other hand, although less explored to date, investigations in human serum samples have recorded a positive correlation between retinol concentrations and the IgA/IgM ratio as an indicator of class switch recombination [19]. In addition, a correlation has been documented between the concentrations of retinol-binding protein 4 (RBP4), which is the main vitamin A transporter protein, and IgA concentrations [20].

Since SIgA is the predominant isoform of this antibody on mucosal surfaces, this study aimed to determine the relationship between the nutritional status of vitamin A, assessed by dietary vitamin A intake and RBP4 concentrations, and SIgA levels in COVID-19 outpatients. The study findings demonstrated a direct correlation of dietary vitamin A intake and RBP4 with SIgA.

## 2. Materials and Methods

### 2.1. Study Design and Patients

Fourteen days post-diagnosis of COVID-19 in the patients, a cross-sectional assessment was carried out. Patients were recruited between January and March 2022 during the critical period for infections in Mexico attributed to the Omicron variant. Patient selection was performed at the Laboratorio de Diagnóstico de Enfermedades Emergentes y Reemergentes (LaDEER) at the Centro Universitario de Ciencias de la Salud (CUCS) de la Universidad de Guadalajara (UdeG). Confirmation of SARS-CoV-2 was established through RT-PCR using the DeCoV19 Kit Triplex assay kit (Genes2Life G2L-DeCoV19-MP, Guanajuato, Mexico). Identification of SARS-CoV-2 variants was also performed using the Master Mut Kit assay (Genes2Life G2LMUSC-14, Guanajuato, Mexico) as previously described [21]. The LaDEER eligibility criteria for RT-PCR testing of suspected COVID-19 patients has previously been published in greater detail [22,23].

The calculation of the sample size was conducted using Cohen’s formula for hypothesis testing of the correlation coefficient [24], wherein the correlation reported by Jones BG et al. [20], served as a reference. A sample size of 33 patients was determined with 80% power and 95% confidence. After confirming COVID-19, people between 18 and 60 years old were invited to participate in the study. Individuals with malabsorption diseases, immunosuppression, autoimmune diseases, lactating or pregnant women, those with a body mass index ≤18.5 kg/m^2^ or ≥39.9 kg/m^2^, and those who had consumed vitamin supplements within the previous two weeks were not included in the study. Patients who consented to participate in the study were provided with an informed consent form that included a thorough description of their participation, possible risks, benefits, and contact details. The current research was conducted in accordance with the Declaration of Helsinki and authorized by the CUCS Research, Research Ethics, and Biosafety Committees under opinion number CI-04621, date of 11 October 2021.

### 2.2. Data Collection

Patients were invited to LaDEER for assessment 14 days after COVID-19 diagnosis. Sociodemographic, clinical data, and vital signs were collected from each patient. The presence of 23 symptoms and their rating according to the Wong–Baker scale were evaluated. Body temperature was assessed utilizing an infrared thermometer (Yuwell YT-1C, Yuwell-Jiangsu Yuyue Medical Equipment & Supply, Shanghai, China), while oxygen saturation was monitored using a digital pulse oximeter (Sonolife LMT-01, Sonolife, Shanghai, China). Heart rate and blood pressure were recorded via a digital sphygmomanometer (Omron HEM-7120, Omron Healthcare, Kyoto, Japan).

### 2.3. Analysis of Serum Samples

Blood sample collection was performed through venipuncture after 8 to 12 h of fasting. A vacuum system was used to draw blood, which was deposited in two 6 mL tubes without anticoagulant. Blood samples were processed for serum separation. Aliquots were prepared and stored at −80 °C until analysis. At all times, precautions were taken to protect the blood and serum samples from direct exposure to light.

Liver function was evaluated by analyzing transaminases through absorbance photometry with BioSystems kits (11531 and 11533, Barcelona, Spain). Additionally, the high-sensitivity C-reactive protein (hs-CRP) concentration as an inflammatory biomarker was determined using BioSystems kit 31927 (Barcelona, Spain) through turbidimetry. Furthermore, the concentration of interleukin 6 (IL-6) was determined using a high-sensitivity sandwich ELISA according to the manufacturer’s instructions (Thermo Fisher Scientific BMS213HS, Dreieich, Germany). In cases where a sample was found to be outside the standard curve, the measurement was repeated in a 1:3 dilution with the kit sample diluent in a volume of 120 μL, and its concentration was multiplied by a dilution factor of three.

The concentration of transforming growth factor beta 1 (TGF-β1) was determined using the BioLegend 436707 kit (San Diego, CA, USA), employing a sandwich ELISA method. The TGF-β1 activation protocol was followed as directed by the manufacturer, although the sample dilution was modified to 1:100 with the kit’s sample diluent in a 300 μL volume. The concentration was multiplied by a dilution factor of 200 in the final calculation.

In addition, the total levels of IgA, IgM, IgG1, IgG2, IgG3, and IgG4 isotypes were determined employing a bead based multiplex immunoassay (Bio-Rad 171A3100M, Hercules, CA, USA), in accordance with the guidelines provided by the manufacturer. The inhibition rate of neutralizing antibodies (NAb) for SARS-CoV-2 (RBD) was determined by a blocking ELISA (GenScript cPass SARS-CoV-2 Neutralization kit (Piscataway, NJ, USA)), following the original protocol.

The concentration of RBP4 was assessed as a surrogate for retinol. For this, a sandwich ELISA (Thermo Fisher Scientific BMS2199, Dreieich, Germany) following the manufacturer’s guidelines was employed. The concentration categories used for classification were as follows: deficiency, <16 μg/mL; insufficiency, 16 to 22 μg/mL; and sufficiency, >22 μg/mL [20,25].

### 2.4. Analysis of Salivary Samples

Each patient was instructed to attend the appointment without having previously exercised, smoked, chewed gum, or drank alcohol or coffee. Patients were asked to have their mouths rinsed with drinking water, and, after a 10 min period, saliva was collected from their mouths. Approximately 2 mL of saliva was collected from each patient in a 50 mL conical tube. To achieve this, patients were instructed to tilt their heads forward, bring the tube to their mouths, and gently guide saliva into the tube until the required volume was reached.

Although 14 days had elapsed since COVID-19 diagnosis, the salivary samples were inactivated based on a previously reported protocol [26]. For this, 10% Triton X-100 (Sigma-Aldrich T8787, Merck KGaA, Darmstadt, Germany) with PBS was applied, and the inactivation reagent was added to each saliva aliquot to achieve a final dilution of 1%. Subsequently, saliva aliquots were incubated with Triton X-100 at room temperature for one hour and stored at −80 °C until analysis.

The salivary SIgA concentration was analyzed using a sandwich ELISA method (Abnova KA3980, Taipei, Taiwan) according to the manufacturer’s instructions. Prior to analysis, the samples were thawed and centrifuged at 3500 rpm for 10 min to remove salivary particles that may have interfered with the assay. The ratio of SIgA to the other isotypes was analyzed to monitor class switching, as previously reported [19].

### 2.5. Assessment of Nutrient Intake

The dietary nutrient intake assessment relied on the 24 h dietary recall (24HR) method of a previous day. The first 24HR was obtained via video call after the patients had consented to participate in the study. The second 24HR was recorded during the evaluation appointment. A photographic food album was employed as a visual support to aid patients in describing their food portions [27]. The food amounts, measured in grams, obtained from both 24HR were entered into the Nutritionist Pro software v7.9 from Axxya Systems (Redmond, WA, USA) to compute the average nutrient intake. The residual method was used to adjust for the total energy intake [28].

The vitamin A dietary intake of each patient was compared with the estimated average requirement (EAR: 500 μg RAE for women; 625 μg RAE for men) and the recommended dietary allowance (RDA: 700 μg RAE for women; 900 μg RAE for men) outlined in the guidelines of the U.S. Institute of Medicine [29].

### 2.6. Statistical Analysis

The data underwent analysis employing the statistical packages IBM SPSS Statistics (version 25.0, Chicago, IL, USA) and GraphPad Prism (version 7.0, San Diego, CA, USA) with a predetermined statistical significance threshold of <0.05. A Shapiro–Wilk test was conducted to assess the normality of the data. The numerical variables were presented using medians with quartiles Q1 and Q3, while categorical variables were expressed as frequencies and percentages.

For between-group comparisons, patients were divided into two groups based on whether they met the EAR for vitamin A (intake below the EAR vs. intake above the EAR). Patients were similarly grouped based on RBP4 concentration, labeled as “insufficient” or “sufficient”. Box-and-whisker plots were used to show the data, displaying medians and interquartile range (Q3–Q1). Comparisons between groups were analyzed using the Mann–Whitney *U* test.

Additionally, a correlation analysis was conducted using Spearman’s correlation coefficient (*rs*). Subsequently, SIgA levels were categorized as “low” or “high” based on median values. An ROC curve analysis was then performed to determine the optimal cutoff value, sensitivity, specificity, and positive likelihood ratio (+LR) of vitamin A intake and RBP4 concentration in discriminating a high SIgA response.

## 3. Results

### 3.1. Clinical, Biochemical, and Nutritional Characteristics

A total of 39 outpatients who had been diagnosed with COVID-19 14 days earlier were assessed. All patients (100%) were infected with the Omicron variant. Among them, COVID-19 was reported to have been experienced for the first time by 84.6% of the patients. Notable persistent symptoms included phlegm (69.2%), cough (64.1%), headache (59%), constipation (48.7%), and weakness (48.7%).

COVID-19 vaccination had already been received by most patients (97.4%). The most frequently used vaccines were Pfizer/BioNTech (31.6%) and CanSino (31.6%). Additionally, it was reported by all vaccinated patients that the complete vaccination schedule had been received. Serum analysis revealed an IL-6 concentration of 0.89 (0.77) pg/mL and an hs-CRP level of 1.73 (3.48) mg/L (Table 1).

The nutritional status of vitamin A was also analyzed in this study. This vitamin was found to be consumed at a level of 511.36 (354.71) μg RAE, with 252.28 (265.47) μg originating from retinol and 111.61 (435.58) μg from carotenoids (Table 2). It was observed that the EAR of vitamin A was not met by 48.8% of the patients, while 79.5% did not reach the RDA of this vitamin. The concentration of RBP4 was 26.77 (17.96) μg/mL. Based on the RBP4 classification in Table 1, it was noted that concentrations below the sufficiency levels were exhibited by 35.9% of patients.

### 3.2. Antibodies by Vitamin A Nutritional Status

It was found that SIgA concentration was 152.4 (90.08) μg/mL. Furthermore, the IgA concentration was 734.96 (325.31) μg/mL. The median inhibition rate of neutralizing antibodies for SARS-CoV-2 (RBD) was 96.8%, as presented in Table 3.

**Figure 1:**
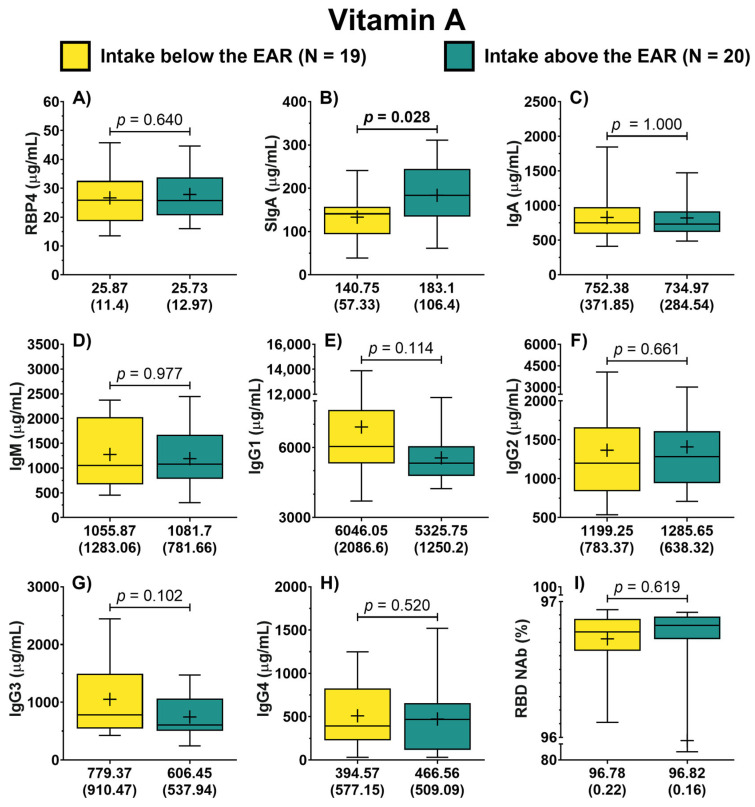
Antibody concentration according to the dietary intake of vitamin A of COVID-19 outpatients. RBP4 levels in patients with either dietary intake below or above the EAR (**A**). The levels of the different antibody isotypes and RBD NAb in patients according to the classification of vitamin A intake (**B**–**I**). The description for each graph includes the median with the interquartile range, Mann–Whitney *U* test. Bold formatting is used to highlight significant *p*-values. Abbreviations: EAR, estimated average requirement; RBP4, retinol-binding protein 4; Ig, immunoglobulin; RBD NAb, receptor-binding domain neutralizing antibodies; hs-CRP, high-sensitivity C-reactive protein; IL-6, interleukin 6; TGF-β1, transforming growth factor beta 1.

Patients were categorized into two groups based on their vitamin A intake, depending on their adherence to the EAR. In this analysis, the concentration of RBP4 was similar between both groups (25.87 vs. 25.73 μg/mL) as depicted in Figure 1A. Similarly, patients with vitamin A intake below the EAR displayed an IgA concentration of 752.38 (371.85) μg/mL, while those with intake above the EAR had a concentration of 734.97 (284.54) μg/mL, evidencing no significant difference (Figure 1C). In contrast, patients with an intake of vitamin A above the EAR showed a significantly higher concentration of SIgA compared to those classified as having an intake of this vitamin below the EAR (183.1 vs. 140.75 μg/mL, *p* = 0.028) (Figure 1B). No differences were found between groups concerning the other isotypes (Figure 1). Regarding the symptom score, patients with vitamin A intake below the EAR reported higher scores than those with vitamin A intake above the EAR (20.5 vs. 10 points); however, this difference was not significant (*p* = 0.095).

**Figure 2:**
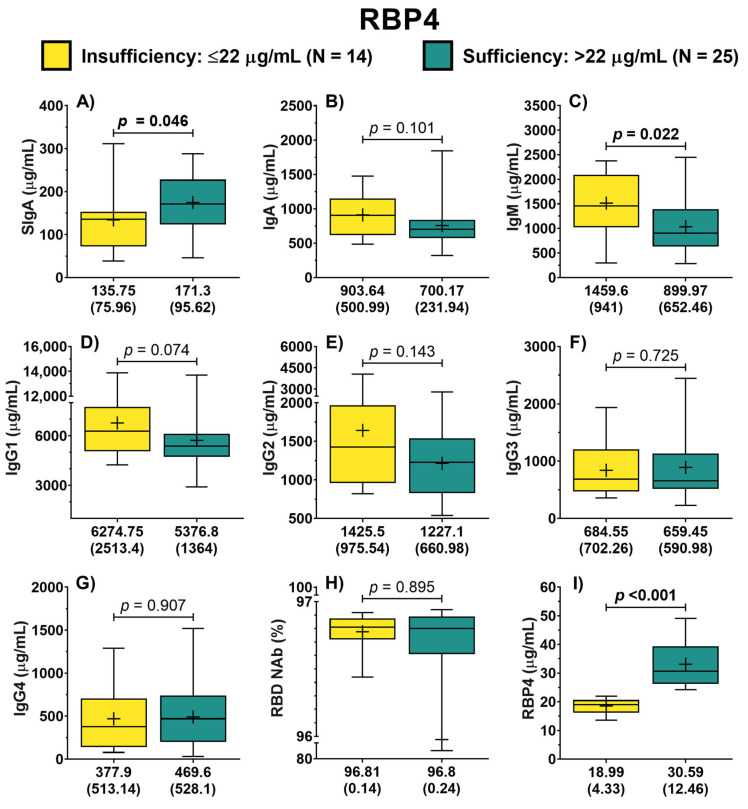
Antibody concentration according to RBP4 classification of COVID-19 outpatients. The levels of the different antibody isotypes and RBD NAb in patients according to the classification of RBP4 concentration (**A**–**H**). RBP4 levels in patients with either RBP4 insufficiency or sufficiency (**I**). The description for each graph includes the median with the interquartile range, Mann–Whitney *U* test. Bold formatting is used to highlight significant *p*-values. Abbreviations: RBP4, retinol-binding protein 4; Ig, immunoglobulin; RBD NAb, receptor-binding domain neutralizing antibodies; hs-CRP, high-sensitivity C-reactive protein; IL-6, interleukin 6; TGF-β1, transforming growth factor beta 1.

Similarly, patients were classified into two groups (insufficiency vs. sufficiency) based on the serum concentration of RBP4. In this analysis, it was possible to identify that the concentration of IgA did not differ between both groups (*p* = 0.101) (Figure 2B). In contrast, patients with sufficiency exhibited higher concentrations of SIgA compared to the group with RBP4 insufficiency (171.3 vs. 135.75 μg/mL, *p* = 0.046) (Figure 2A). Furthermore, it was identified that patients with RBP4 sufficiency displayed lower concentrations of IgM (*p* = 0.022) (Figure 2C). Also, patients with RBP4 sufficiency showed a trend of lower IgG1 concentrations compared to patients with insufficiency (*p* = 0.074) (Figure 2D). In the case of symptoms score, no difference in the rating between both groups was identified (12.5 vs. 14 points, *p* = 0.639).

### 3.3. Association of Vitamin A Nutritional Status with SIgA

**Figure 3:**
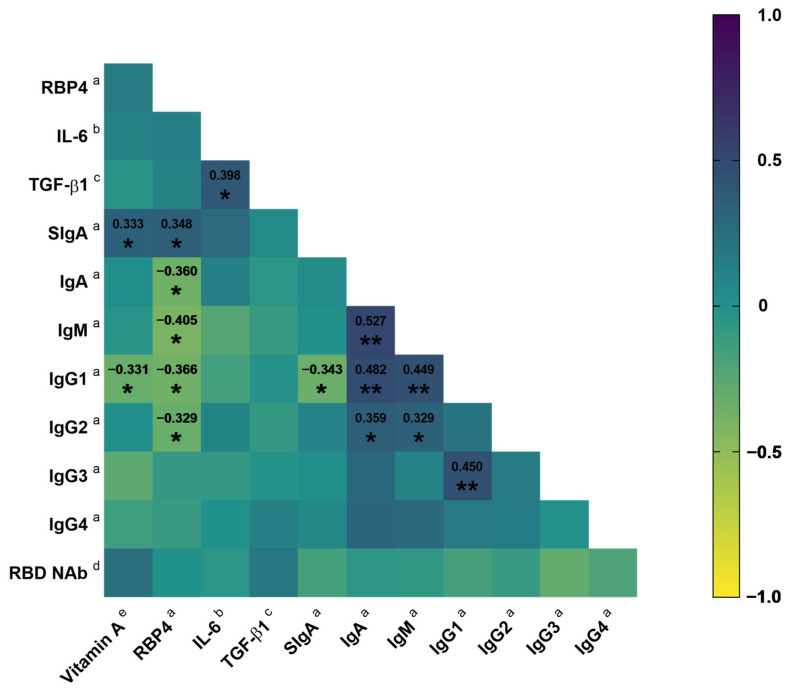
Correlation matrix between vitamin A intake and RBP4 with the antibody concentration in COVID-19 outpatients. Spearman correlation coefficient. * *p* < 0.05; ** *p* < 0.01. ^a^ μg/mL; ^b^ pg/mL; ^c^ ng/mL; ^d^ %; ^e^ μg RAE. Abbreviations: RBP4, retinol-binding protein 4; IL-6, interleukin 6; TGF-β1, transforming growth factor beta 1; Ig, immunoglobulin; RBD NAb, receptor-binding domain neutralizing antibodies.

In the correlation analysis, it was observed that dietary intake of vitamin A exhibited a positive correlation with RBP4 (*rs* = 0.155, *p* = 0.351) and RBD NAb (*rs* = 0.236, *p* = 0.154); however, these correlations were not significant. In contrast, there was a significant positive correlation between the intake of this vitamin and SIgA (*rs* = 0.333, *p* = 0.041). Furthermore, the consumption of vitamin A exhibited a negative correlation with IgG1 (*rs* = −0.331, *p* = 0.042). Additionally, RBP4 exhibited a positive correlation with SIgA (*rs* = 0.348, *p* = 0.030), while displaying negative correlations with IgA (*rs* = −0.360, *p* = 0.024), IgM (*rs* = −0.405, *p* = 0.011), IgG1 (*rs* = −0.366, *p* = 0.022), and IgG2 (*rs* = −0.329, *p* = 0.041). Concerning IgG1, it negatively correlated with SIgA (*rs* = −0.343, *p* = 0.033) (Figure 3). In addition, vitamin A intake showed a trend of negative correlation with symptom score (*rs* = −0.298, *p* = 0.069). Likewise, RBP4 was negatively correlated with symptom score; however, it was not significant (*rs* = −0.149, *p* = 0.364).

Similarly, vitamin A intake showed a positive correlation with the SIgA/IgG1 ratio (*rs* = 0.405, *p* = 0.012) and SIgA/IgG3 ratio (*rs* = 0.405, *p* = 0.012). Furthermore, a positive correlation was found between RBP4 and the SIgA/IgM ratio (*rs* = 0.477, *p* = 0.002), SIgA/IgG1 ratio (*rs* = 0.426, *p* = 0.007), and SIgA/IgG2 ratio (*rs* = 0.517, *p* = 0.001). Although, with the SIgA/IgG3 ratio, it only exhibited a positive correlation tendency (*rs* = 0.284, *p* = 0.080). Likewise, days elapsed from symptom onset to the time of evaluation exhibited a positive correlation with SIgA (*rs* = 0.333, *p* = 0.039), SIgA/IgM (*rs* = 0.386, *p* = 0.015), SIgA/IgG1 (*rs* = 0.345, *p* = 0.032), and SIgA/IgG2 (*rs* = 0.376, *p* = 0.021).

Additionally, an ROC curve analysis was conducted to identify the cutoff values for dietary vitamin A intake and RBP4 in discriminating high SIgA concentrations in the evaluated population. In this analysis, it was identified that patients with a dietary vitamin A intake greater than 476 μg RAE (AUC = 0.706 [CI 95%: 0.538–0.873]) had a 2.22 likelihood ratio of having a high SIgA concentration (*p* = 0.031). Furthermore, patients with an RBP4 concentration above 25 μg/mL (AUC = 0.763 [CI 95%: 0.613–0.914]) exhibited a 2.26 likelihood ratio of having a high SIgA concentration (*p* = 0.005). These cutoff values displayed a sensitivity of 77.8% and 78.9%, respectively (Table 4).

## 4. Discussion

The results of the current study demonstrate a significant prevalence of suboptimal vitamin A intake and insufficient RBP4 concentrations in COVID-19 outpatients. However, both vitamin A intake and RBP4 concentrations were found to be directly correlated with SIgA. Furthermore, patients with higher vitamin A consumption exhibited a higher SIgA/IgG1 and SIgA/IgG3 ratio, while those with higher RBP4 concentrations showed a higher SIgA/IgM, SIgA/IgG1, and SIgA/IgG2 ratio.

Vitamin A deficiency remains among the top nutritional deficiencies worldwide, especially in developing populations [30]. Nutritional transition in recent decades has been characterized by the consumption of ultra-processed foods [31]. The intake of such food has been associated with a decreased provision of essential micronutrients for human health, such as vitamin A [32]. In a report derived from the Encuesta Nacional de Salud y Nutrición (National Health and Nutrition Survey) 2012 in the Mexican population, it was observed that a diet based on ultra-processed food results in a 50% lower intake of vitamin A compared to a diet based on unprocessed or minimally processed food [33]. Likewise, previous research in the Mexican population has reported a high prevalence of insufficient vitamin A intake [22,34,35], which is in accordance with the present study.

The leading cause of vitamin A deficiency is insufficient intake of retinol and carotenoid food sources [11,14]. RBP4 is often used as a surrogate for retinol to determine vitamin A nutritional status [20,36]. However, both retinol concentration and RBP4 synthesis can be compromised in acute inflammatory processes, as demonstrated in patients hospitalized for COVID-19 [37,38,39]. According to previous reports, the acute-phase response of mild COVID-19 has a duration of approximately 5 to 9 days [40,41]. In the present study, more than one-third of the patients had insufficient RBP4 concentration even though the time elapsed, at the time of evaluation, had already passed the acute phase of infection.

In addition to contributing to visual health, vitamin A has fundamental functions in the immune system response [11,14]. Studies have reported that low intake and low serum concentration of vitamin A have been associated with respiratory tract infections [42,43]. Furthermore, according to Sirisinha et al. [15], Wistar rats assigned to a vitamin A-deficient diet exhibited lower concentrations of SIgA in intestinal samples compared to the control group. The previously mentioned is similar to what was reported in the present study, where patients with vitamin A intake below the EAR and insufficient RBP4 concentrations had lower SIgA levels. As discussed in previous reviews, RA—an active metabolite of vitamin A—can act alone and in combination with other stimuli to promote germinal centers, resulting in enhanced antibody response, especially of the IgA class [44]. More recently, it has been suggested that RA, in conjunction with the retinoic acid receptor (RAR) and retinoid X receptor (RXR), bind directly to the α-switch region of the Ig heavy chain gene to facilitate activation-induced cytidine deaminase (AID) activity to promote class switching to IgA antibodies [12].

The above has been observed predominantly in GALT; however, several preclinical studies have shown that mice inoculated intranasally with a respiratory virus and subsequently assigned to a vitamin A-rich diet or vitamin A supplementation exhibit a better SIgA response compared to the control group [16,17,18]. In addition, intranasal retinol administration improved the SIgA response in nasal washes in vitamin A-deficient mice inoculated with a respiratory virus [45]. In this regard, it has been proposed that vitamin A also has a close relationship with the SIgA response in the respiratory tract for airborne virus infection prevention. According to Rudraraju et al. [46], aldehyde dehydrogenase 1 (ALDH1A), which is involved in the metabolism of retinal to RA, is constitutively expressed in upper and lower respiratory tract epithelial cells. They also described that these cells could produce cytokines that, in synergy with RA, over-regulate IgA production in this tissue [46].

Consistent with the above, a direct correlation of vitamin A and RBP4 with SIgA was identified in the present study. SIgA in this study was analyzed from salivary samples. As has been reviewed in other studies, nasal-associated lymphoid tissue (NALT) corresponds to the inductive site of IgA^+^ plasmablasts that migrate to the salivary glands, where plasmablasts differentiate into IgA-producing plasma cells [47]. However, the formation of ectopic germinal centers in the salivary glands has also been proposed [48]. Likewise, it has been suggested that GALT contributes significantly to the expansion and class switch to IgA^+^ plasmablasts, which then migrate to the salivary glands. The migration of plasmablasts is controlled by their expression of integrins and chemokine receptors [49,50,51]. The previously mentioned factors could explain the positive correlation between dietary intake of vitamin A and RBP4 with salivary SIgA identified in the present study, since vitamin A is essential in the production of IgA in GALT [12], and retinol could be directly metabolized in the epithelial cells of NALT to favor class switching [46].

Although it is generally accepted that the mucosal and systemic immune systems act separately in the humoral response [47], studies in patients immunized against SARS-CoV-2 intramuscularly have shown anti-SARS-CoV-2 antibodies of the SIgA class in saliva samples [8,52]. Exactly how intramuscular administration of vaccines induces the mucosal SIgA response is not known at this time; however, it has been suggested that vaccination may generate a mucosal IgA response in the intestine and that plasma cells generated in the intestine may migrate into the oral cavity [52]. Similarly, it has been suggested that B cells from peripheral lymph nodes could mobilize to the mucosa under the influence of their homing receptors to differentiate into plasma cells and secrete SIgA [8,53]. In the present study, a positive correlation was identified between vitamin A intake and the SIgA/IgG1 and SIgA/IgG3 ratio. In turn, RBP4 was positively correlated with the SIgA/IgM, SIgA/IgG1, and SIgA/IgG2 ratios. Therefore, considering that nearly all evaluated patients had been previously immunized with SARS-CoV-2 vaccines, it is hypothesized that vitamin A may have promoted class switching at the systemic level, leading to the production of IgA^+^ plasmablasts which could be subsequently mobilized to the site of infection for SIgA production.

According to a previous report, RBP4 correlated with IgA, IgA/IgM, and IgA/IgG1 in serum samples [20]; however, in the present study, these correlations were not replicated. In fact, RBP4 correlated negatively with serum IgA. The above could support the hypothesis that after the class switching, there is an increased migration of IgA^+^ plasmablasts to effector sites in the airways to favor SIgA production. However, it should not rule out the possibility that RBP4 could be redistributed to the infection sites to directly promote the release of retinol, which, when metabolized, favors SIgA production in the salivary glands since, in the present study, a positive correlation of RBP4 with SIgA was identified.

Furthermore, an exploratory analysis was performed to determine the correlation of vitamin A nutritional status with COVID-19 symptomatology; however, no significant correlations of dietary vitamin A intake and RBP4 levels with symptom score were identified. Likewise, SIgA did not correlate with symptom score. Thus, the potential protective role of SIgA against COVID-19 symptomatology in outpatients was not established in the present study.

To our understanding, this is the first study in which both dietary vitamin A intake and RBP4 concentrations are evaluated together to determine their correlation with SIgA in human biological samples. In addition, the samples were collected within the period when SIgA kinetics peak after a natural respiratory tract infection. However, in the present study, it was not possible to assess salivary SIgA specific to SARS-CoV-2 because there was no kit available. This assay could have provided valuable information since it corresponds directly to SIgA with neutralizing activity. Also, all patients had already been exposed to the antigen (vaccinated or previous COVID-19), so they could have mounted an immune response different from the primary response, which could also have masked the correlation of IL-6 and TGF-β1 with SIgA. On the other hand, apo-RBP4 is found in circulation, although to a much lesser extent than holo-RBP4 (15% vs. 85%) [36]; therefore, it is considered relevant to include in other studies the analysis of retinol directly as a biomarker of vitamin A nutritional status.

Previously, meeting the daily RDA of vitamin A has been proposed as a prophylactic intervention for respiratory infections. In addition, in cases of mild COVID-19, the administration of mega doses of 60,000 μg of RAE (200,000 IU) of vitamin A for two consecutive days has been suggested to promote antibody response [54], which is similar to measles treatment in children [13]. According to previous studies, the use of RA as a vaccine adjuvant has been suggested [55,56,57], which could be crucial in second-generation oral and nasal vaccine formulation for SARS-CoV-2 and other emerging and reemerging viruses. Likewise, the stabilization of sufficient retinol concentrations before respiratory virus seasonality, either through a healthy diet or supplementation, could be an appropriate strategy for respiratory tract infection prevention.

## 5. Conclusions

In conclusion, dietary vitamin A intake and RBP4 concentration were correlated with SIgA. Furthermore, patients with higher vitamin A intake exhibited a higher SIgA/IgG1 and SIgA/IgG3 ratio, whereas those with higher RBP4 concentrations showed higher SIgA/IgM, SIgA/IgG1, and SIgA/IgG2 ratios. Therefore, although the present study cannot establish causality, it is suggested that nutritional vitamin A status may be a pivotal contributor to the SIgA response in preventing the incidence and progression of respiratory infections.

## Figures and Tables

**Table 1:** Clinical and biochemical characteristics of COVID-19 outpatients.

Variable	*N* = 39
Age (years)	35 (29–52)
Sex, *n* (%)	
Female	23 (59)
Male	16 (41)
Time since onset of symptoms (days)	19 (17–20)
Vaccine administered, *n* (%)	
Pfizer/BioNTech	12 (31.6)
AstraZeneca/Oxford	8 (21.1)
Sputnik V	1 (2.6)
Sinovac	2 (5.3)
CanSino	12 (31.6)
Moderna	3 (7.9)
Full vaccination schedule, *n* (%)	38 (100)
Time since last vaccination (days)	134 (59–194)
Symptoms (score)	14 (5.5–24)
Temperature (°C)	36.4 (36.35–36.6)
Respiratory rate (rpm)	14 (14–17)
Oxygen saturation (SpO_2_)	96 (95–98)
Heart rate (ppm)	74 (68–81)
SBP (mmHg)	115 (110–142)
DBP (mmHg)	79 (73–86)
AST/GOT (U/L)	23.2 (19.39–30.66)
ALT/GPT (U/L)	20.32 (14.45–29.41)
hs-CRP (mg/L)	1.73 (0.39–3.87)
IL-6 (pg/mL)	0.89 (0.44–1.21)
TGF-β1 (ng/mL)	19.75 (15.75–25.52)
RBP4 (μg/mL)	26.77 (19.61–37.57)
RBP4 classification, *n* (%)	
Deficiency	2 (5.1)
Insufficiency	12 (30.8)
Sufficiency	25 (64.1)

Data presented in medians (Q1–Q3). Abbreviations: SBP, systolic blood pressure; DBP, diastolic blood pressure; AST/GOT, aspartate aminotransferase; ALT/GPT, alanine aminotransferase; hs-CRP, high-sensitivity C-reactive protein; IL-6, interleukin 6; TGF-β1, transforming growth factor beta 1; RBP4, retinol-binding protein 4.

**Table 2:** Dietary intake of nutrients of COVID-19 outpatients.

Variable	*N* = 39
Lipids (g)	73.48 (58.89–84.29)
Vitamin A (μg RAE)	511.36 (323.19–677.9)
Vitamin D (μg)	3.42 (1.72–6.01)
Vitamin E (mg)	3.6 (3.01–5.71)
Vitamin K (μg)	25.25 (18.59–45.65)
Magnesium (mg)	275.32 (217.9–350.11)
Zinc (mg)	9.71 (7.75–11.41)
Retinol (μg)	252.28 (172.09–437.56)
Carotenoids (μg)	111.61 (39.98–475.56)
β-carotene (μg)	93.81 (38.21–383.01)
α-carotene (μg)	5.94 (1.91–43.09)
β-cryptoxanthin (μg)	5.42 (0.98–17.84)

Data presented in medians (Q1–Q3). Dietary nutrient intake was adjusted for total energy intake using the residual method.

**Table 3:** Antibody concentration of COVID-19 outpatients.

Variable	*N* = 39
SIgA (μg/mL)	152.4 (120.72–210.8)
IgA (μg/mL)	734.96 (613.32–938.63)
IgM (μg/mL)	1065.5 (696.15–1685.1)
IgG1 (μg/mL)	5574.4 (4780.85–6696.25)
IgG2 (μg/mL)	1262.2 (907.87–1586.45)
IgG3 (μg/mL)	659.45 (514.89–1130.55)
IgG4 (μg/mL)	436.26 (191.53–702.08)
RBD NAb (%)	96.8 (96.7–96.88)

Data presented in medians (Q1–Q3). Abbreviations: Ig, immunoglobulin; RBD NAb, receptor-binding domain neutralizing antibodies.

**Table 4:** Positive likelihood ratios of dietary vitamin A and RBP4 cut-off values to high SIgA.

Variable	Cut-Off	+LR (CI 95%)	Sensitivity (CI 95%)	Specificity (CI 95%)	*p*-Value
Vitamin A	>476 μg RAE	2.22 (1.16–4.24)	77.8 (54.8–91.0)	65 (43.3–81.9)	**0.031**
RBP4	>25.05 μg/mL	2.26 (1.19–4.28)	78.9 (56.7–91.5)	65 (43.3–81.9)	**0.005**

Abbreviations: +LR, positive likelihood ratio; CI, confidence intervals. Bold formatting is used to highlight significant *p*-values.

## Data Availability

The data described in the manuscript are available on request from the corresponding author.

## References

[1] Jin X., Ren J., Li R., Gao Y., Zhang H., Li J., Zhang J., Wang X., Wang G. (2021). Global Burden of Upper Respiratory Infections in 204 Countries and Territories, from 1990 to 2019. eClinicalMedicine.

[2] Safiri S., Mahmoodpoor A., Kolahi A.-A., Nejadghaderi S.A., Sullman M.J.M., Mansournia M.A., Ansarin K., Collins G.S., Kaufman J.S., Abdollahi M. (2023). Global Burden of Lower Respiratory Infections during the Last Three Decades. Front. Public Health.

[3] Calderaro A., Buttrini M., Farina B., Montecchini S., De Conto F., Chezzi C. (2022). Respiratory Tract Infections and Laboratory Diagnostic Methods: A Review with A Focus on Syndromic Panel-Based Assays. Microorganisms.

[4] Polatoğlu I., Oncu-Oner T., Dalman I., Ozdogan S. (2023). COVID-19 in Early 2023: Structure, Replication Mechanism, Variants of SARS-CoV-2, Diagnostic Tests, and Vaccine & Drug Development Studies. MedComm.

[5] Bonanni P., Ceddia F., Dawson R. (2023). A Call to Action: Current Challenges and Considerations for COVID-19 Vaccination in Immunocompromised Populations. J. Infect. Dis..

[6] Velikova T., Snegarova V., Kukov A., Batselova H., Mihova A., Nakov R. (2021). Gastrointestinal Mucosal Immunity and COVID-19. World J. Gastroenterol..

[7] Li Y., Jin L., Chen T. (2020). The Effects of Secretory IgA in the Mucosal Immune System. BioMed Res. Int..

[8] Sano K., Bhavsar D., Singh G., Floda D., Srivastava K., Gleason C., Carreño J.M., Simon V., Krammer F., PARIS Study Group (2022). SARS-CoV-2 Vaccination Induces Mucosal Antibody Responses in Previously Infected Individuals. Nat. Commun..

[9] Wang Z., Lorenzi J.C.C., Muecksch F., Finkin S., Viant C., Gaebler C., Cipolla M., Hoffmann H.-H., Oliveira T.Y., Oren D.A. (2021). Enhanced SARS-CoV-2 Neutralization by Dimeric IgA. Sci. Transl. Med..

[10] Russell M.W., Mestecky J. (2022). Mucosal Immunity: The Missing Link in Comprehending SARS-CoV-2 Infection and Transmission. Front. Immunol..

[11] Carazo A., Macáková K., Matoušová K., Krčmová L.K., Protti M., Mladěnka P. (2021). Vitamin A Update: Forms, Sources, Kinetics, Detection, Function, Deficiency, Therapeutic Use and Toxicity. Nutrients.

[12] Bos A., Van Egmond M., Mebius R. (2022). The Role of Retinoic Acid in the Production of Immunoglobulin A. Mucosal Immunol..

[13] Huiming Y., Chaomin W., Meng M., The Cochrane Collaboration (2005). Vitamin A for Treating Measles in Children. Cochrane Database of Systematic Reviews.

[14] Amimo J.O., Michael H., Chepngeno J., Raev S.A., Saif L.J., Vlasova A.N. (2022). Immune Impairment Associated with Vitamin A Deficiency: Insights from Clinical Studies and Animal Model Research. Nutrients.

[15] Sirisinha S., Darip M.D., Moongkarndi P., Ongsakul M., Lamb A.J. (1980). Impaired Local Immune Response in Vitamin A-Deficient Rats. Clin. Exp. Immunol..

[16] Cui D., Stephensen C.B., Moldoveanu Z. (2000). High-Level Dietary Vitamin A Enhances T-Helper Type 2 Cytokine Production and Secretory Immunoglobulin A Response to Influenza A Virus Infection in BALB/c Mice. J. Nutr..

[17] Surman S.L., Jones B.G., Sealy R.E., Rudraraju R., Hurwitz J.L. (2014). Oral Retinyl Palmitate or Retinoic Acid Corrects Mucosal IgA Responses toward an Intranasal Influenza Virus Vaccine in Vitamin A Deficient Mice. Vaccine.

[18] Surman S.L., Penkert R.R., Jones B.G., Sealy R.E., Hurwitz J.L. (2016). Vitamin Supplementation at the Time of Immunization with a Cold-Adapted Influenza Virus Vaccine Corrects Poor Mucosal Antibody Responses in Mice Deficient for Vitamins A and D. Clin. Vaccine Immunol..

[19] Patel N., Penkert R.R., Sealy R.E., Surman S.L., Jones B.G., Ringwald-Smith K., Ross A.C., Hurwitz J.L. (2022). Retinol Binding Protein, Sunlight Hours, and the Influenza Virus-Specific Immune Response. Biomedicines.

[20] Jones B.G., Oshansky C.M., Bajracharya R., Tang L., Sun Y., Wong S.S., Webby R., Thomas P.G., Hurwitz J.L. (2016). Retinol Binding Protein and Vitamin D Associations with Serum Antibody Isotypes, Serum Influenza Virus-Specific Neutralizing Activities and Airway Cytokine Profiles. Clin. Exp. Immunol..

[21] Muñoz-Valle J.F., Venancio-Landeros A.A., Sánchez-Sánchez R., Reyes-Díaz K., Galindo-Ornelas B., Hérnandez-Monjaraz W.S., García-Ríos A., García-Ortega L.F., Hernández-Bello J., Peña-Rodríguez M. (2022). An Upgrade on the Surveillance System of SARS-CoV-2: Deployment of New Methods for Genetic Inspection. Int. J. Mol. Sci..

[22] González-Estevez G., Turrubiates-Hernández F.J., Herrera-Jiménez L.E., Sánchez-Zuno G.A., Herrera-Godina M.G., Muñoz-Valle J.F. (2021). Association of Food Intake Quality with Vitamin D in SARS-CoV-2 Positive Patients from Mexico: A Cross-Sectional Study. Int. J. Environ. Res. Public Health.

[23] Macedo-Ojeda G., Muñoz-Valle J.F., Yokogawa-Teraoka P., Machado-Sulbarán A.C., Loza-Rojas M.G., García-Arredondo A.C., Tejeda-Constantini R., Vega-Magaña A.N., González-Estevez G., García-Chagollán M. (2021). COVID-19 Screening by Anti-SARS-CoV-2 Antibody Seropositivity: Clinical and Epidemiological Characteristics, Comorbidities, and Food Intake Quality. Int. J. Environ. Res. Public Health.

[24] Machin D., Campbell M.J., Tan S.B., Tan S.H. (2008). Sample Size Tables for Clinical Studies.

[25] De Pee S., Dary O. (2002). Biochemical Indicators of Vitamin A Deficiency: Serum Retinol and Serum Retinol Binding Protein. J. Nutr..

[26] Isho B., Abe K.T., Zuo M., Jamal A.J., Rathod B., Wang J.H., Li Z., Chao G., Rojas O.L., Bang Y.M. (2020). Persistence of Serum and Saliva Antibody Responses to SARS-CoV-2 Spike Antigens in COVID-19 Patients. Sci. Immunol..

[27] Vizmanos-Lamotte B., López-Uriarte P.J., Hunot-Alexander C., Bernal-Orozco M.F., Rodríguez-Rocha N.P., Macedo-Ojeda G., Martínez-Lomelí L., Rovillé-Sausse F. (2015). Álbum Fotográfico de Alimentos Mexicanos.

[28] Willett W., Howe G., Kushi L. (1997). Adjustment for Total Energy Intake in Epidemiologic Studies. Am. J. Clin. Nutr..

[29] Institute of Medicine (U.S.) Committee to Review Dietary Reference Intakes for Vitamin D and Calcium (2011). Dietary Reference Intakes for Vitamin D and Calcium.

[30] Zhao T., Liu S., Zhang R., Zhao Z., Yu H., Pu L., Wang L., Han L. (2022). Global Burden of Vitamin A Deficiency in 204 Countries and Territories from 1990–2019. Nutrients.

[31] Popkin B.M., Ng S.W. (2022). The Nutrition Transition to a Stage of High Obesity and Noncommunicable Disease Prevalence Dominated by Ultra-processed Foods Is Not Inevitable. Obes. Rev..

[32] Martini D., Godos J., Bonaccio M., Vitaglione P., Grosso G. (2021). Ultra-Processed Foods and Nutritional Dietary Profile: A Meta-Analysis of Nationally Representative Samples. Nutrients.

[33] Marrón-Ponce J.A., Sánchez-Pimienta T.G., Rodríguez-Ramírez S., Batis C., Cediel G. (2023). Ultra-processed Foods Consumption Reduces Dietary Diversity and Micronutrient Intake in the Mexican Population. J. Hum. Nutr. Diet..

[34] Pedroza-Tobías A., Hernández-Barrera L., López-Olmedo N., García-Guerra A., Rodríguez-Ramírez S., Ramírez-Silva I., Villalpando S., Carriquiry A., Rivera J.A. (2016). Usual Vitamin Intakes by Mexican Populations. J. Nutr..

[35] Ramírez-Silva I., Rodríguez-Ramírez S., Barragán-Vázquez S., Castellanos-Gutiérrez A., Reyes-García A., Martínez-Piña A., Pedroza-Tobías A. (2020). Prevalence of Inadequate Intake of Vitamins and Minerals in the Mexican Population Correcting by Nutrient Retention Factors, Ensanut 2016. Salud Pública México.

[36] Tanumihardjo S.A., Russell R.M., Stephensen C.B., Gannon B.M., Craft N.E., Haskell M.J., Lietz G., Schulze K., Raiten D.J. (2016). Biomarkers of Nutrition for Development (BOND)—Vitamin A Review. J. Nutr..

[37] Mohd M.A., Ahmad Norudin N.A., Muhammad T.S.T. (2020). Transcriptional Regulation of Retinol Binding Protein 4 by Interleukin-6 via Peroxisome Proliferator-Activated Receptor α and CCAAT/Enhancer Binding Proteins. Mol. Cell. Endocrinol..

[38] Tepasse P.-R., Vollenberg R., Fobker M., Kabar I., Schmidt H., Meier J.A., Nowacki T., Hüsing-Kabar A. (2021). Vitamin A Plasma Levels in COVID-19 Patients: A Prospective Multicenter Study and Hypothesis. Nutrients.

[39] Vollenberg R., Tepasse P.-R., Fobker M., Hüsing-Kabar A. (2022). Significantly Reduced Retinol Binding Protein 4 (RBP4) Levels in Critically Ill COVID-19 Patients. Nutrients.

[40] Kirtana J., Kumar A., Kumar S., Singh A., Shankar S., Sharma A., Kumar A., Kaur R., Khan M., Ranjan P. (2020). Mild COVID-19 Infection-Predicting Symptomatic Phase and Outcome: A Study from AIIMS, New Delhi. J. Fam. Med. Prim. Care.

[41] Liu J., Li S., Liu J., Liang B., Wang X., Wang H., Li W., Tong Q., Yi J., Zhao L. (2020). Longitudinal Characteristics of Lymphocyte Responses and Cytokine Profiles in the Peripheral Blood of SARS-CoV-2 Infected Patients. eBioMedicine.

[42] Abdelkader A., Wahba A.A., El-tonsy M., Zewail A.A., Shams Eldin M. (2022). Recurrent Respiratory Infections and Vitamin A Levels: A Link? It Is Cross-Sectional. Medicine.

[43] Almoosawi S., Palla L. (2020). Association between Vitamin Intake and Respiratory Complaints in Adults from the UK National Diet and Nutrition Survey Years 1–8. BMJ Nutr. Prev. Health.

[44] Ross A.C., Chen Q., Ma Y. (2011). Vitamin A and Retinoic Acid in the Regulation of B-Cell Development and Antibody Production. Vitamins & Hormones.

[45] Surman S.L., Jones B.G., Rudraraju R., Sealy R.E., Hurwitz J.L. (2014). Intranasal Administration of Retinyl Palmitate with a Respiratory Virus Vaccine Corrects Impaired Mucosal IgA Response in the Vitamin A-Deficient Host. Clin. Vaccine Immunol..

[46] Rudraraju R., Jones B.G., Surman S.L., Sealy R.E., Thomas P.G., Hurwitz J.L. (2014). Respiratory Tract Epithelial Cells Express Retinaldehyde Dehydrogenase ALDH1A and Enhance IgA Production by Stimulated B Cells in the Presence of Vitamin A. PLoS ONE.

[47] Bemark M., Angeletti D. (2021). Know Your Enemy or Find Your Friend?—Induction of IgA at Mucosal Surfaces. Immunol. Rev..

[48] Grewal J.S., Pilgrim M.J., Grewal S., Kasman L., Werner P., Bruorton M.E., London S.D., London L. (2011). Salivary Glands Act as Mucosal Inductive Sites *via* the Formation of Ectopic Germinal Centers after Site-restricted MCMV Infection. FASEB J..

[49] Jackson D.E., Lally E.T., Nakamura M.C., Montgomery P.C. (1981). Migration of IgA-Bearing Lymphocytes into Salivary Glands. Cell. Immunol..

[50] Czerkinsky C., Svennerholm A.M., Quiding M., Jonsson R., Holmgren J. (1991). Antibody-Producing Cells in Peripheral Blood and Salivary Glands after Oral Cholera Vaccination of Humans. Infect. Immun..

[51] Kunkel E.J., Butcher E.C. (2003). Plasma-Cell Homing. Nat. Rev. Immunol..

[52] Sheikh-Mohamed S., Isho B., Chao G.Y.C., Zuo M., Cohen C., Lustig Y., Nahass G.R., Salomon-Shulman R.E., Blacker G., Fazel-Zarandi M. (2022). Systemic and Mucosal IgA Responses Are Variably Induced in Response to SARS-CoV-2 mRNA Vaccination and Are Associated with Protection against Subsequent Infection. Mucosal Immunol..

[53] Su F., Patel G.B., Hu S., Chen W. (2016). Induction of Mucosal Immunity through Systemic Immunization: Phantom or Reality?. Hum. Vaccines Immunother..

[54] Midha I.K., Kumar N., Kumar A., Madan T. (2021). Mega Doses of Retinol: A Possible Immunomodulation in COVID-19 Illness in Resource-limited Settings. Rev. Med. Virol..

[55] Ma Y., Ross A.C. (2005). The Anti-Tetanus Immune Response of Neonatal Mice Is Augmented by Retinoic Acid Combined with Polyriboinosinic:Polyribocytidylic Acid. Proc. Natl. Acad. Sci. USA.

[56] Lisulo M.M., Kapulu M.C., Banda R., Sinkala E., Kayamba V., Sianongo S., Kelly P. (2014). Adjuvant Potential of Low Dose All- *Trans* Retinoic Acid during Oral Typhoid Vaccination in Zambian Men. Clin. Exp. Immunol..

[57] Said D.E., Amer E.I., Sheta E., Makled S., Arafa F.M., Diab H.E. (2023). Nano-Encapsulated Antioxidant: Retinoic Acid as a Natural Mucosal Adjuvant for Intranasal Immunization against Chronic Experimental Toxoplasmosis. Trop. Med. Infect. Dis..

